# Overview of distinct 5-methylcytosine profiles of messenger RNA in human hepatocellular carcinoma and paired adjacent non-tumor tissues

**DOI:** 10.1186/s12967-020-02417-6

**Published:** 2020-06-22

**Authors:** Qiyao Zhang, Qingyuan Zheng, Xiao Yu, Yuting He, Wenzhi Guo

**Affiliations:** 1 Department of Hepatobiliary and Pancreatic Surgery, The First Affiliated Hospital of Zhengzhou University, No.1 Jianshe Road, Zhengzhou, 450052 Henan China; 2 Key Laboratory of Hepatobiliary and Pancreatic Surgery and Digestive Organ Transplantation of Henan Province, The First Affiliated Hospital of Zhengzhou University, Zhengzhou, 450052 China; 3 Open and Key Laboratory of Hepatobiliary & Pancreatic Surgery and Digestive Organ Transplantation at Henan Universities, Zhengzhou, 450052 China; 4 Henan Key Laboratory of Digestive Organ Transplantation, Zhengzhou, 450052 China

**Keywords:** mRNA, 5-methylcytosine, Hepatocellular carcinoma, RNA methylation, MeRIP-seq

## Abstract

**Background:**

Post-transcriptional methylation modifications, including 5-methylcytosine (m5C) modification, are closely related to the tumorigenesis of cancers. However, the mRNA profile of m5C modification in hepatocellular carcinoma (HCC) is unknown.

**Methods:**

Methylated RNA immunoprecipitation sequencing was performed to identify m5C peaks on mRNA of human HCC tissues and adjacent tissues, and differences in m5C between the two groups were analyzed. In addition, we conducted a bioinformatics analysis to predict the function of specific methylated transcripts.

**Results:**

We found that there was a noticeable difference in m5C between HCC and paired non-tumor tissues, suggesting that m5C could play a role in the pathogenesis of HCC. In addition, analyses of gene ontology and the Kyoto Encyclopedia of Genes and Genomes showed that the unique distribution pattern of mRNA m5C in HCC was associated with a wide range of cellular functions.

**Conclusions:**

Our results revealed different distribution patterns of m5C in HCC and adjacent tissues and provided new insights into a novel function of m5C RNA methylation of mRNA in HCC progression.

## Background

Hepatocellular carcinoma (HCC) is one of the most widespread cancers, and it has an extremely poor prognosis, contributing to nearly 662,000 deaths per annum [1, 2]. The incidence rate of HCC is ranked sixth-highest of the cancerous tumors globally, and the number of deaths caused by HCC is ranked third-highest of the tumor-related deaths [3]. Despite marked progress in treatment, due to its late diagnosis, high metastasis, and high recurrence rate, the lethal rate of hepatocellular carcinoma remains high [4–7]. In addition to the widely used alpha-fetoprotein (AFP), protein induced by vitamin K absence or antagonist-II (PIVKA-II), and third electrophoretic form of lentil lectin-reactive AFP (AFPL3) tumor markers for HCC, some miRNAs and new biomarkers, such as heat shock protein 90-α (Hsp90α) and a metabolite biomarker, have been discovered recently, and have shown high performance in the diagnosis of HCC [8–11]. Nevertheless, the early diagnosis of hepatocellular carcinoma still requires further investigation. Personalized immunotherapy based on immunophenotyping has become a research hotspot in recent years [6, 12], but the stability and effectiveness of this model is still uncertain. Therefore, a deeper understanding of the pathogenesis of HCC and the identification of new biomarkers are essential for early diagnosis and developing new therapeutic targets of HCC.

Epigenetic dysregulation plays a critical role in the initiation and progression of cancer, and post-transcriptional modifications, such as RNA methylation, have attracted the attention of many researchers [13]. With advances in high-throughput technologies, such as m5C-RNA immunoprecipitation (IP), researchers have been able to characterize RNA methylation sites in-depth [14]. Increasing evidence has shown that N6-methyladenosine (m6A), which is the most prevalent internal mRNA modification, is related to mRNA metabolism, such as regulating mRNA stability and splicing [15–19]. In addition, related studies have shown the potential molecular mechanism in cancer, including HCC. Li et al. found that inhibiting the generation of YTHDTF2, which is an m6A reader protein, blocked anti-miR-145-enhanced proliferation, suggesting that miR-145 suppresses the proliferation of HCC cells by regulating m6A reading [20, 21]. 5-methylcytosine (m5C), which is another post-transcriptional RNA modification, has been identified in stable and highly abundant tRNAs, rRNAs, and mRNAs [22–24]. In addition, NSUN2 has been identified as a methyltransferase, while ALYREF and YBX1 have been identified as an m5C reader [25]. Studies have shown that m5C modification is necessary for the stable and efficient translation of tRNA and plays an important role in rRNA processing, structuring, and translation [26–29]. This modification has conservative, tissue-specific, and dynamic characteristics in mammalian transcriptomes [25]. Thus, a study on mice demonstrated that m5C is primarily enriched near the translation initiation codon of the embryonic stem cells and brain of mice [20]. However, this feature has not been found in Arabidopsis [30], and the distribution characteristics of m5C could be different for different cell types. Chen et al. [31] demonstrated that m5C can promote the pathogenesis of bladder cancer by stabilizing mRNAs. However, the quantity, distribution, and functions of m5C in HCC are still unclear.

We performed a m5C-specific analysis and in-depth bioinformatics analysis of m5C in mRNA in human HCC and paired adjacent non-tumor tissues. The results showed marked differences in the amount and distribution of m5C between HCC and adjacent tissues: the number of m5C methylation peaks in HCC was much more than that in paired adjacent non-tumor tissues, and the difference in distribution was wide and involved all chromosomes. Bioinformatics analysis showed that the two groups, with different methylation, could cause different changes in cell function. Our findings suggest a possible association between HCC and m5C in mRNA and predict possible functional changes caused by this difference in m5C.

## Materials and methods

### RNA extraction and fragmentation

Each pair of HCC tissues and paired adjacent non-tumor tissues were obtained from the First Affiliated Hospital of Zhengzhou University. We collected six groups of biological replicates (Table 1). Subsequently, we extracted total RNA using TRIzol reagent (Invitrogen Corporation, CA, USA) following the manufacturer’s instructions. A Ribo-Zero rRNA Removal Kit (Illumina, Inc., CA, USA) was used to reduce the rRNA content. The quality of RNA was evaluated using its OD260/OD280 ratio, which is a measure of the nucleotide to protein ratio based on optical density measured using spectrophotometry. The purity of RNA with an OD260/OD280 value range of 1.8–2.1 was considered acceptable, and the RNA extracted from all samples met this standard.

**Table 1:** Clinical characteristics of HCC patients

Age	Gender	AFP (ng/mg)	Stage (BCLC)	Tumor size (cm)	Tumor metastasis	HBsAG	HBcAb
47	Male	2.19	A1	7*5.5*4	Right lobe of liver	–	+
45	Male	614.1	A1	3.8*3.3*2.7	No	+	+
60	Male	4602	A2	6*5.3*3.5	No	+	+
49	Male	191.7	A1	3.5*2.5*2	No	+	+
78	Male	2.83	A3	4.5*4*2	No	+	+
45	Male	1.69	A2	2.5*2*2	No	+	+

### Library construction and sequencing

Methylated RNA immunoprecipitation sequencing (MeRIP-seq) was performed based on a previously reported procedure [32]. Total RNA was lysed into 100 base pair fragments using a GenSeqTM m5C RNA IP Kit (GenSeq Inc, China), and m5C immunoprecipitation was performed followed by RNA-seq library generation using a NEBNext^®^ Ultra II Directional RNA Library Prep Kit (New England Biolabs, Inc, USA). A BioAnalyzer 2100 system (Agilent Technologies, Inc, USA) was used to evaluate the cDNA library, and library sequencing was performed using an Illumina Hiseq instrument with 150 bp paired-end reads.

### Identification and analysis of 5-methylcytosine peaks

Quality control of the paired-end reads was performed using a quality standard of the probability of an incorrect base call at 1 in 1000 (Q30) in Illumina HiSeq 4000 Sequencer: a Q30 > 80% indicated good sequencing quality (Table 2). After conducting quality control, the 3′ adaptors were trimmed and low-quality reads were removed using Cutadapt software (v1.9.3), and high-quality clean reads were harvested. Clean reads of input libraries were aligned to a reference genome (GRCh38.gencode. v32) using STAR software [33] and mRNA peaks were identified using DCC software [34]. Next, clean reads of all libraries were aligned to the reference genome using Hisat2 software (v2.0.4) [35]. Then, the m5C peaks on the mRNA were identified using MACS software [36]. In addition, differentially methylated peaks were identified using DiffReps software [37]: m5C peaks with a fold change > 2 or < 0.5 (*P* value ≤ 0.00001) in HCC were considered to be up-regulated methylation or down-regulated methylation. Peaks identified using both software and the section of m5C that overlapped with the exon of the protein-coding genes were selected using scripts developed in-house for further annotation.

**Table 2:** The Q30 of simples

Patient ID	Sample	Q30	Sample	Q30
1	HCC IP	92.39%	HCC input	92.91%
2	HCC IP	92.86%	HCC input	94.25%
3	HCC IP	93.04%	HCC input	94.49%
4	HCC IP	93.28%	HCC input	94.03%
5	HCC IP	93.26%	HCC input	91.90%
6	HCC IP	92.17%	HCC input	94.04%
1	Paired non-tumor IP	91.86%	Paired non-tumor input	93.04%
2	Paired non-tumor IP	91.77%	Paired non-tumor input	92.96%
3	Paired non-tumor IP	91.55%	Paired non-tumor input	93.81%
4	Paired non-tumor IP	90.36%	Paired non-tumor input	94.10%
5	Paired non-tumor IP	91.16%	Paired non-tumor input	89.47%
6	Paired non-tumor IP	90.86%	paired non-tumor input	92.47%

### Statistical analysis

The m5C peaks on the mRNA of the six samples in the HCC group were combined to obtain the m5C peaks of the HCC group, and the adjacent tissue group was treated in the same way. Bedtools software was used to find common peaks between the two groups. The sequence of methylated peaks, which was 50 bp on each side of the apex, was scanned using Dreme software [38] to find meaningful motif. The E-values for the motifs were calculated as the enrichment P-value times the number of candidate motifs tested, and the enrichment P-value was calculated using Fisher’s Exact Test for the enrichment of the motif in the positive sequences. The lower the E-value of the motif, the higher its credibility. Subsequently, the methylation fold enrichment (FE) of each mRNA of the six pairs of samples was acquired and subjected to log2 conversion. The logFE value was used for clustering in the heatmap.2 software package. In addition, we counted the mRNA region where the m5C peak was located in each sample according to published methods [39] and plotted the results as a pie chart.

### Transcriptome sequencing analysis

High-quality reads were mapped onto the genome (human gencode v32) using hisat2 software (v2.0.4) [35]. Then, HTSeq software (v0.9.1) was used to obtain gene-level raw counts as mRNA expression profiles [40]. Edge R software (v3.16.5) was used to normalize the data and calculate the fold change and P-value between the two groups of samples to screen for differentially expressed mRNA [41]. Subsequently, the expression levels of these mRNAs were normalized to obtain logCPM and the average values for the two groups were calculated. A scatterplot of differential expression of methylated genes was draw combining the methylation degree of these genes.

### Bioinformatics analysis

**Fig. 1:**
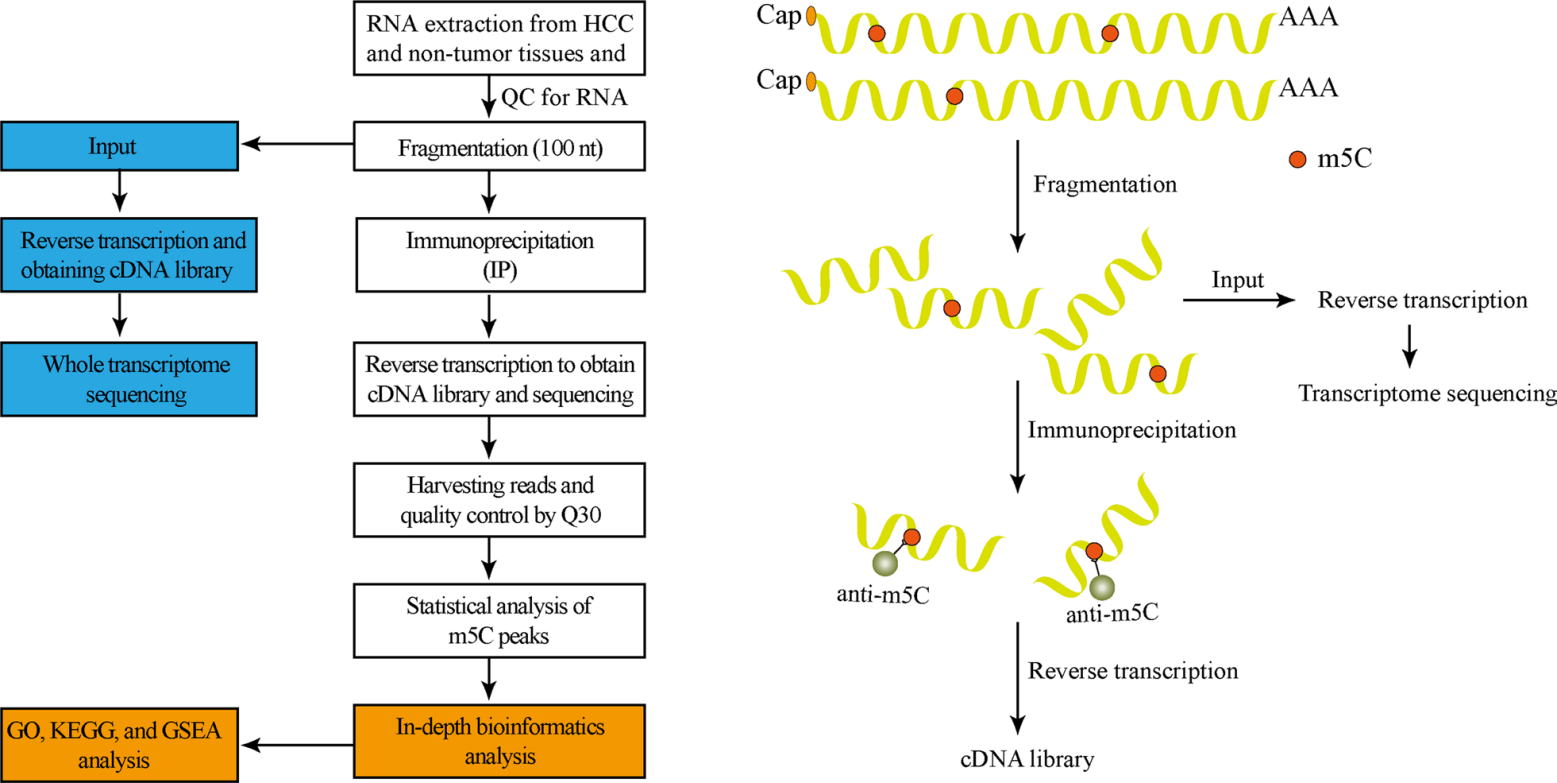
Flowchart of the study

The Gene Ontology (GO) project, which is a structured, controlled vocabulary developed for annotating genes and gene products [42], contains three parts: biological processes (BP), molecular functions (MF), and cellular components (CC). Differentially methylated genes were used to perform GO functional analysis (http://www.geneontology.org) to annotate and speculate on the function of these differentially methylated genes. Gene terms with a *P*-value < 0.05 were considered statistically significant. Meanwhile, pathway analysis using the Kyoto Encyclopedia of Genes and Genomes (KEGG) (https://david.ncifcrf.gov/)was conducted with differentially methylated genes to annotate and speculate pathways in which they could be involved. The pathways with *P*-values < 0.05 were considered significantly enriched. In addition, we used the enrichment intensity fold change value of the two groups of samples in the MeRIP-seq experiment to rank all the coded genes with signals, and we used the PreRanked mode of gene set enrichment analysis (GSEA) to determine the over-representation and under-representation of all genes in a gene set to observe the differences in the gene sets between the two groups. We selected FDR < 0.25 as the screening criterion. We also analyzed other GSEA pathways, with a primary focus on TGF-beta signaling, hedgehog signaling pathway, axon guidance, regulation of actin cytoskeleton, and the arrhythmogenic right ventricular cardiomyopathy arc. All processes are described in Fig. 1.

## Results

### General features of 5-methylcytosine methylation in human HCC and adjacent tissues

**Fig. 2:**
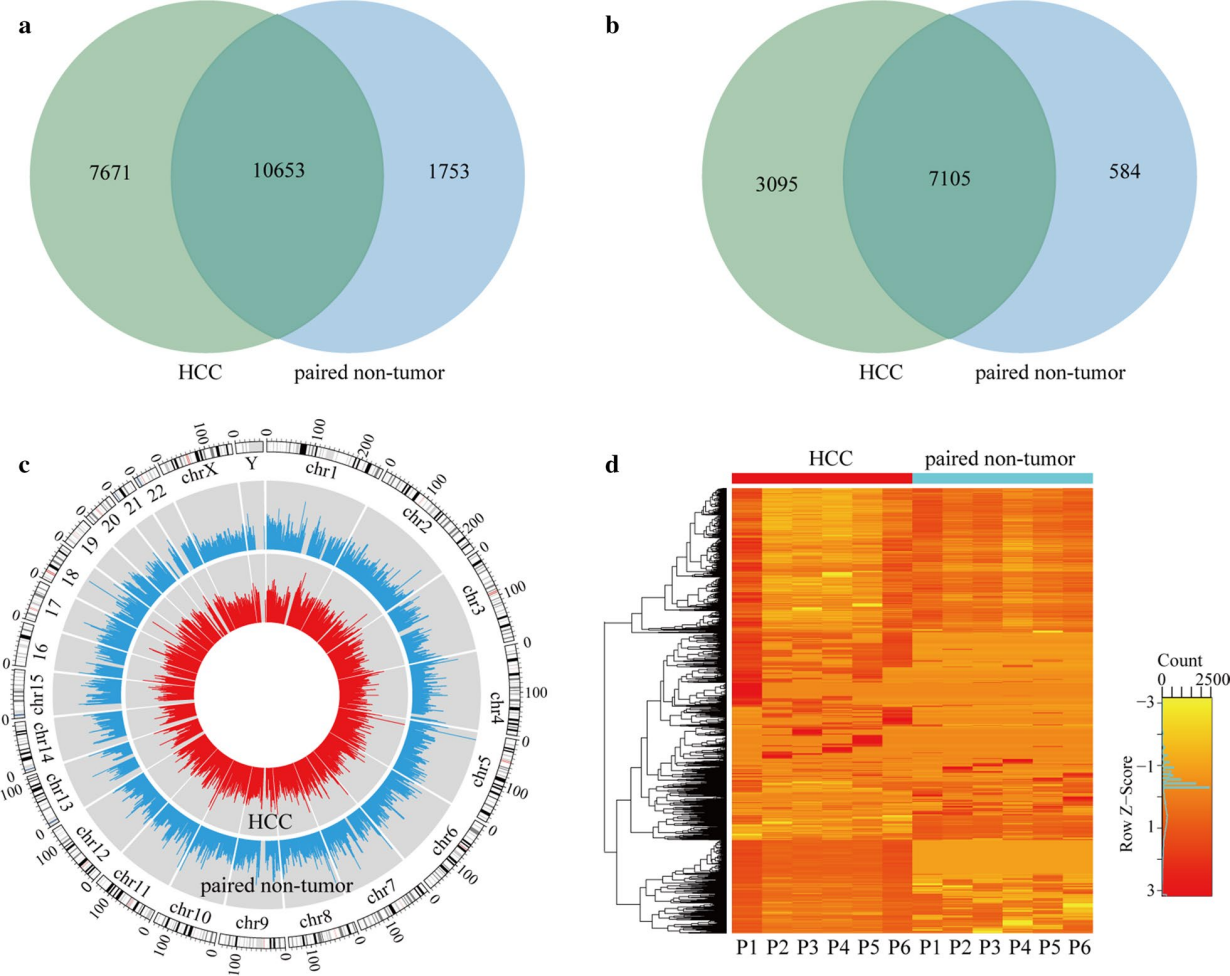
Overview of mRNA 5-methylcytosine (m5C) in hepatocellular carcinoma (HCC) and adjacent tissues. **a** Venn diagram of m5C peaks in HCC and adjacent tissues. **b** Venn diagram of m5C genes in HCC and adjacent tissues. **c** Visualization of m5C at the chromosome level in HCC and adjacent tissues. **d** Cluster analysis of m5C in HCC and adjacent tissues. The color represents the size of the logfold enrichment (FE) value: the closer the color is to red, the larger the logFE value

In general, we found 18,324 clean methylation peaks in HCC tissues and 12,406 clean methylation peaks in adjacent tissues. We mapped up to 10,200 annotated genes of HCC tissues and 7689 annotated genes of adjacent tissues. Of 18,324 methylation peaks, only 7671 appeared in HCC, while only 1753 of 12,406 methylation peaks appeared in adjacent tissues. (Figure 2a, b). There were noticeable differences in the number of m5C peaks and differentially methylated m5C peaks. Moreover, for the methylation peaks that only appeared in HCC or adjacent tissues, the number of up-methylated peaks per gene in HCC tissues (2.48 peaks/gene) was smaller than that in adjacent tissues (3.00 peaks/gene), and both of them were markedly higher than the methylated peaks that were present in both samples (1.50 peaks/gene).

When the distribution of mRNA m5C peaks on the chromosomes was analyzed using circos software, it was found that the number and distribution of m5C peaks on each chromosome were different between HCC and adjacent tissues, with the difference on chromosome one being the most obvious (Fig. 2c). In addition, compared with the sex chromosomes, the autosomes in both groups were more methylated.

### Cluster analysis

The results of the methylation heatmap and cluster analyses showed that clustering of methylation differences could clearly distinguish the HCC group from the adjacent tissues group: there were relative consistencies within the groups and marked differences between the groups (Fig. 2d). Overall, the degree of methylation in the HCC tissues was higher than in the adjacent tissues. In total, 3126 methylation peaks in HCC were identified as up-regulated methylation, and 1103 methylation peaks were detected as down-regulated methylation (Tables 3 and 4).

**Table 3:** Top ten up-methylated peaks

Chromosome	TxStart	TxEnd	Gene name	Fold change
17	44,802,641	44,803,000	GJC1	166.3
20	20,368,103	20,368,560	INSM1	107.8
8	87,871,361	87,871,880	DCAF4L2	105.5
1	27,395,481	27,395,814	GPR3	53.6
8	2,001,041	2,001,420	KBTBD11	49.958333
6	130,438,081	130,438,480	TMEM200A	46.512195
11	109,427,881	109,428,300	C11orf87	41.321429
1	14,924,221	14,924,540	KAZN	40.7
X	37,728,401	37,728,740	XK	39.196721
9	16,416,641	16,417,340	BNC2	38.307692

**Table 4:** Top ten down-methylated peaks

Chromosome	TxStart	TxEnd	Gene name	Fold change
11	75,083,541	75,084,020	OR2AT4	139.3
11	48,325,401	48,325,840	OR4C3	134.4
10	17,137,441	17,137,840	TRDMT1	123.9
11	4,490,301	4,490,760	OR52K1	113.9
11	102,713,021	102,713,457	MMP8	109.1
8	53,225,801	53,226,240	OPRK1	108.9
14	62,109,501	62,109,960	SYT16	101.4
X	50,367,561	50,368,020	DGKK	93.3
11	5,069,601	5,070,240	OR52E1	92.6
Y	5,740,441	5,740,880	PCDH11Y	92.5

### Motif analysis

**Fig. 3:**
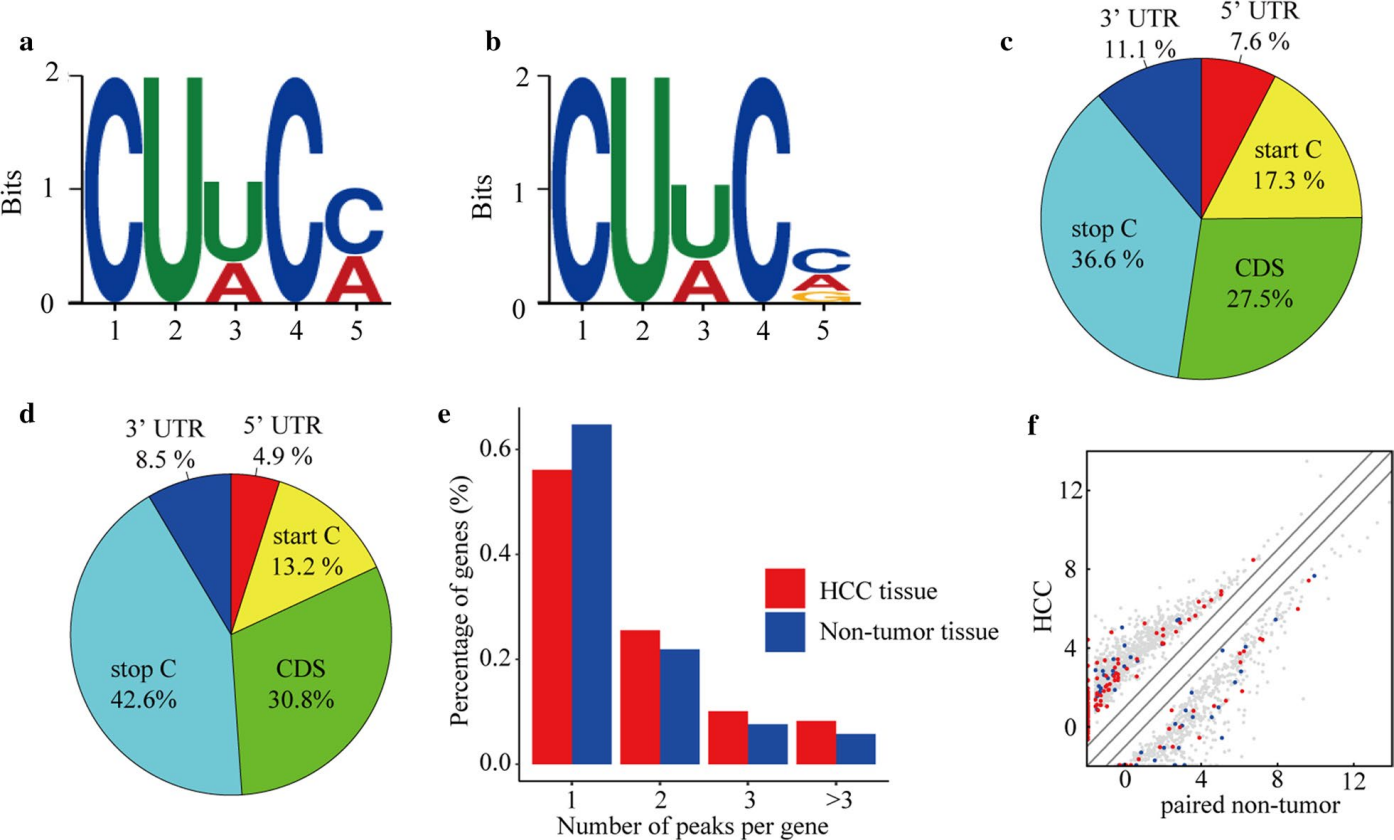
The characteristics of m5C peaks and the joint analysis of methylation and transcriptome. **a** Motif with minimum *E*-value of m5C in the hepatocellular carcinoma (HCC) group. **b** Motif with minimum *E*-value of m5C in the adjacent tissues group. These two motifs are only slightly different at the last base. **c, d** Pie chart of m5C peaks in different regions of mRNA. **e** The number of m5C peaks in HCC and adjacent tissues on each mRNA. Most mRNAs have only one methylation peak. **f** Scatter plot of the relationship between gene expression level and methylation level. The Y-axis and X-axis represent the expression levels of mRNAs in HCC and paired non-tumor tissues, red dots represent mRNAs with a high methylation level, blue dots represent low methylation horizontal mRNAs, and gray represents no significant difference

Among the motifs measured in the HCC tissues, CUWCM (M = C/A) was the most common and reliable: it was the most likely conserved methylation site motif, with an *E*-value of 4.6e-059 (Fig. 3a, b). The motif with the lowest *E*-value in the adjacent tissue was CUWCV, with an *E*-value of 4.4e-067 (V = C/A/G) (Fig. 3a, b). These two motifs were only slightly different at the last base.

### Analysis of sources of mRNA methylation in hepatocellular carcinoma and adjacent tissues

The results of the analysis of the source of methylation peaks showed that m5C was distributed in all regions of the mRNA (Fig. 3c, d), which is consistent with previous research results [15, 43, 44], and the distribution in HCC was similar to that in the adjacent tissues (Fig. 3c, d). Specifically, m5C is mostly distributed near the stop codon and least distributed at 5′UTR in both groups, which could be related to the length of each region on the mRNA. Compared with adjacent tissues, m5C in HCC was more distributed at both the 3′UTR (HCC: 11.1%, adjacent tissues: 8.5%) and 5′UTR (HCC: 7.6%, adjacent tissues: 4.9%) and less distributed near the stop codon (HCC: 36.6%, adjacent tissues: 42.6%).

### Statistical analysis of the number of peaks on each mRNA

When we counted the number of m5C peaks on each mRNA in the two groups, we found that most of the methylated mRNAs (56.1%) in the HCC tissues had only one m5C peak, while this index in adjacent tissues was significantly higher (64.7%, p < 0.0001; Fig. 3e). In addition, the number of mRNAs with two or more m5C peaks on one mRNA was higher in HCC tissue than in adjacent tissues (p < 0.0001).

### Effect of methylation on transcriptional expression

Consistent with the above results, the joint analysis of the methylation and transcriptome data showed that there were more up-regulated methylated mRNAs in the HCC group (Fig. 3f). Among the mRNAs that were up-methylated in HCC, 125 mRNAs were up-regulated, and 38 mRNAs were down-regulated. Among the mRNAs that were up-methylated in adjacent tissues, a total of 47 mRNAs were up-regulated, and 48 mRNAs were down-regulated. mRNAs with higher degrees of methylation tended to have higher expression levels in the HCC group, while this trend was not evident in the adjacent tissues. Moreover, we found that some mRNAs that are rarely expressed in adjacent tissues were highly expressed and hypermethylated in HCC tissues.

### Bioinformatics analysis

**Fig. 4:**
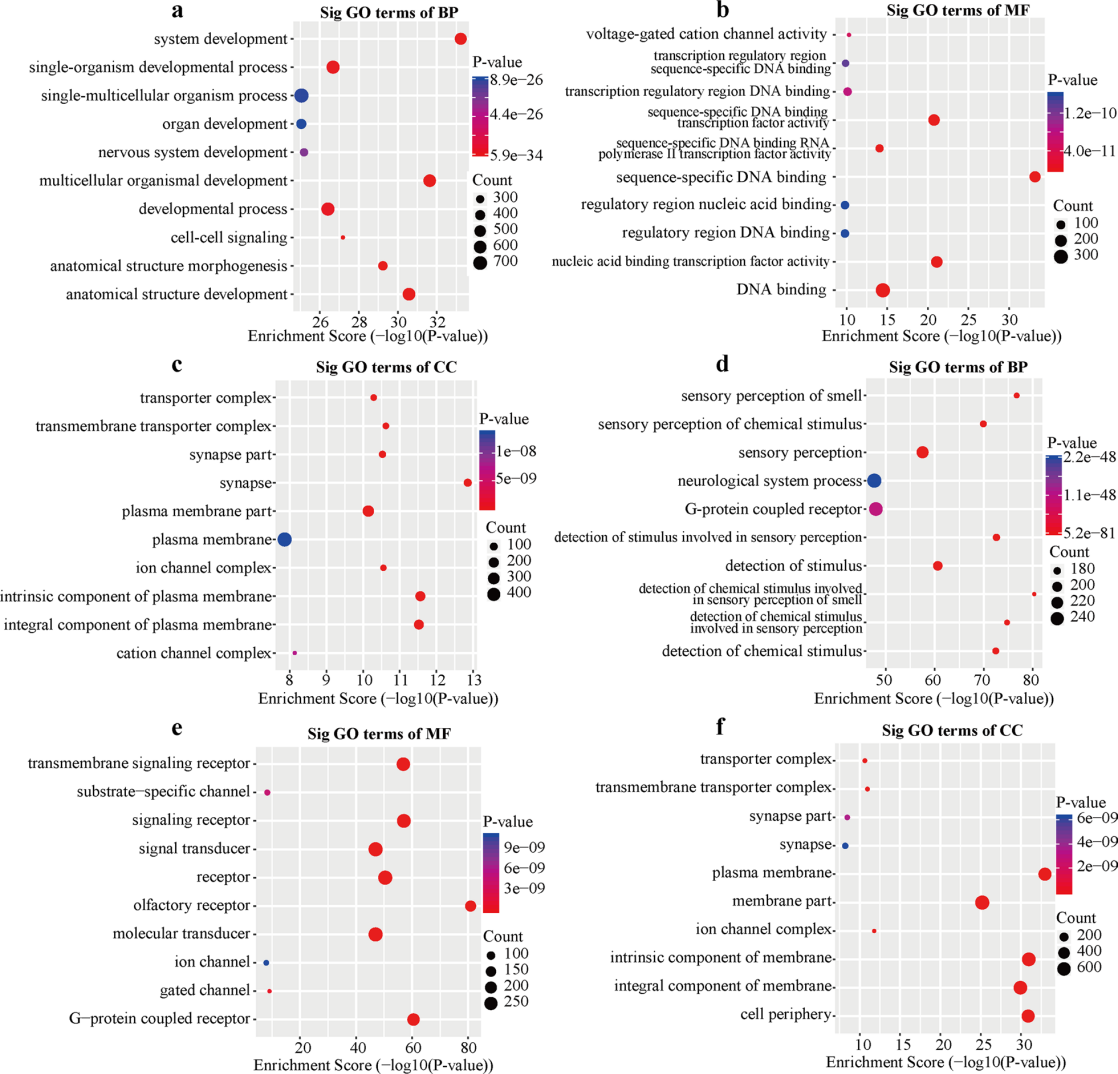
Gene ontology analyses of hepatocellular carcinoma (HCC) and adjacent tissues. **a** biological processes (BP), **b** molecular functions (MF), and **d** cell component (CC) in the HCC group. **c** biological processes, **e,** molecular functions, and **f** cell component in the adjacent tissues group. We have listed the 10 most significant terms in each figure

**Fig. 5:**
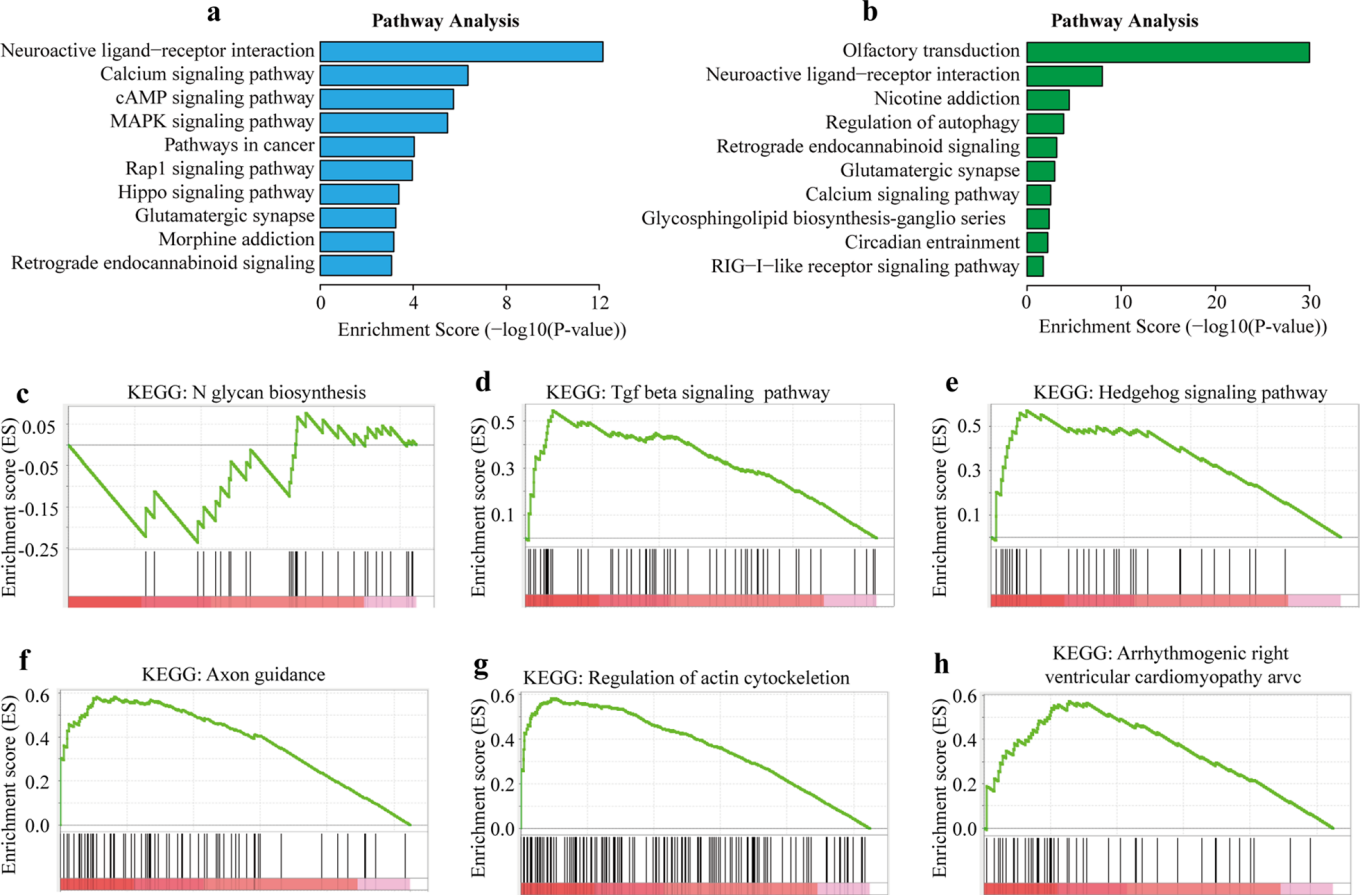
Kyoto Encyclopedia of Genes and Genomes (KEGG) analysis and gene set enrichment analysis (GSEA) of differentially methylated genes in hepatocellular carcinoma (HCC) and adjacent tissues. **a** Pathway analysis of up-methylated genes in the HCC group. **b** Pathway analysis of up-methylated genes in the adjacent tissues group. We have listed the 10 most significant terms on each figure. **c** Down-regulated pathway in the GESA. **d–h** up-regulated pathways in the GESA

The GO analysis of BP found that the genes with up-methylated m5C sites in HCC tissues were significantly enriched in system development, multicellular organismal development, and anatomical structure development (Fig. 4a), while the genes with down-methylated m5C were significantly enriched in the detection of chemical stimuli involved in the sensory perception of smell (Fig. 4d). The GO analysis of MF found that the genes with up-methylated m5C in HCC tissues were mainly related to sequence − specific DNA binding and nucleic acid binding transcription factor activity (Fig. 4b), while the genes with down-methylated m5C were primarily related to olfactory receptor activity and G − protein coupled receptor activity (Fig. 4e). The GO analysis of CC found that the genes with up-methylated m5C in HCC tissues were primarily enriched in the synapse, intrinsic, and integral components of plasma membrane (Fig. 4c), which was the same as the genes with up-methylated m5C in adjacent cancer tissues (Fig. 4f). The KEGG analysis results showed that the mRNAs with up-methylation in HCC tissues were primarily involved in the neuroactive ligand − receptor interaction, calcium signaling pathway, and cAMP signaling pathway (Fig. 5a). The mRNAs with down-methylated m5C were significantly enriched in olfactory transduction, neuroactive ligand − receptor interaction, and nicotine addiction (Fig. 5b).

### Gene set enrichment analysis

We identified 144 gene sets that were up-regulated and five that were down-regulated in HCC tissues compared with the adjacent tissues. In addition, we identified a particularly down-regulated gene set, KEGG N-glycan biosynthesis (Fig. 5c). When we analyzed other GSEA pathways, focusing on TGF-beta signaling, hedgehog signaling pathway, axon guidance, regulation of actin cytoskeleton, and arrhythmogenic right ventricular cardiomyopathy arc (all p values < 0.05) we found that these pathways are related to the pathogenesis and invasiveness of HCC (Fig. 5d–h) [45–47].

## Discussion

As a post-transcriptional modification, RNA methylation has gradually been shown to be involved in many cellular functions and cancers. Studies have shown that multiple types of RNA methylation (m5C and m2G) can stabilize RNA fragments in sperm and thus contribute to the identity of sperm RNA as an epigenetic information carrier [48–50]. Research by Li et al. [21, 51] proved that m6A RNA were effective in 33 different types of cancer, including HCC. For example, they found that increased methylation of m6A mRNA is a carcinogenic mechanism of hepatoblastoma (HB). In addition, METTL3 significantly up-regulates HB and promotes the development of HB, and CTNNB1 has been identified as a regulatory factor for METTL3 to guide m6A to modify HB. As a novel methylation, m5C has been confirmed to play a critical role in regulating RNA nucleation [25], intergenerational transmission of acquired phenotypes [52], and stabilizing mRNAs to promote the proliferation of bladder cancer [31]. To date, the distribution and function of m5C in hepatocellular carcinoma have not been studied.

We sequenced m5C peaks of mRNA in HCC and adjacent tissues using MeRIP-seq and analyzed the differences between the two groups. We identified tens of thousands of m5C peaks in mRNAs and observed significant differences between the two groups. The degree of methylation of mRNA in HCC was significantly higher than in adjacent tissues. In addition, the number of mRNAs mapped using methylation peaks in HCC was markedly higher than in adjacent tissues, which indicates that the role of m5C in HCC tissues is more extensive than in adjacent tissues. This finding was also supported by the cluster analysis, in which we found that the two groups can be clearly distinguished by FE of m5C. The FE of m5C in HCC was significantly higher than that in adjacent tissues, especially for some specific mRNAs. As the function of m5C is not completely clear, we can only make some suggestions: m5C could play a role in promoting the pathogenesis of HCC through its similar mechanism in bladder cancer, i.e., by stabilizing these mRNAs. The motif analysis also showed that the most credible motif in the two groups was very similar, indicating that the differences in m5C in the two groups could be due to differences in the number of methylases rather than the category of methylases. This hypothesis could guiding further experiments.

Numerous studies have shown that the distribution of methylation sites in different regions of the mRNA is essential for the stability of mRNA and the regulation of translation. Dominissini et al.’s study found that m1A is significantly enriched near the start of translation in mammalian and yeast cells and that m1A is associated with higher protein expression [53, 54]. Recent studies showed that in eukaryotes, m5C regulates translation in a negative way [55, 56], which could also work through the m5C sites near the start codon. Our results show that the peaks of m5C located in the start codon in HCC are significantly higher than those in the adjacent tissues. Our findings indicate that the expression of some proteins will be reduced, resulting in the deletion of some essential proteins. In addition, we found that there are fewer m5C peaks at the 3′UTR in HCC, which could also cause differences in cell functions. For example, m5C at the 3′UTR may affect the binding of miRNA or RBPs to mRNA and thus regulate the translation process [43]. This mechanism has not been proven in HCC, so more sophisticated experiments are needed for further validation.

Research by Huang et al. [57] found that abnormal N-glycosylation of proteins is involved in the development of malignant tumors, including HCC. Interestingly, GSEA of differentially methylated genes in our study showed a significant decrease in N-glycan biosynthesis in HCC tissues. N-glycans are major constituents of glycoproteins in eukaryotes and play an important role in many protein functions, such as protein folding and stability [58]. Abnormal glycosylation caused by the up-regulation of fucosyltransferase is closely related to the malignant behavior of the proliferation of HCC [59]. This gene set was significantly down-regulated in HCC (FDR = 0.208), which could suggest that m5C promotes glycosylation disorders in HCC and causes increased malignant behaviors of HCC tissues. The bioinformatics analysis of GO terms and KEGG pathways showed that m5C was involved in various aspects of cell function. Although we have a preliminary understanding of the functions of m5C, such as regulating RNA nuclei [25], affecting cell differentiation [60], regulating stem cell function, and stress [61], most of its mechanisms of action, especially in cancer, are still unknown. Therefore, there is a need for more research to establish a detailed understanding of the role of m5C. Our research provides a new perspective for later researchers to study the role of m5C in HCC to discover more information on the new therapeutic targets of HCC.

## Conclusions

Our research reveals differences in m5C in mRNA of hepatocellular carcinoma and adjacent tissues for the first time and shows the distribution and possible function of m5C through statistical analysis and in-depth bioinformatics analysis. We showed that m5C has a wide range of functions. However, its role in cells, especially in cancer, remains largely unknown. Therefore, more research is needed to determine its roles.

## Data Availability

The datasets used and/or analyzed during the current study are available from the corresponding author on reasonable request.

## Abbreviations

m5C: 5-Methylcytosine
miRNA: micro RNA
HCC: Hepatocellular carcinoma
ncRNAs: Non-coding RNAs
m6A: N6-Methyladenosine
m1A: N1-Methyladenosine
GO: Gene ontology
KEGG: Kyoto Encyclopedia of Genes and Genomes
GSEA: Gene set enrichment analysis
BP: Biological processes
MF: Molecular functions
CC: Cellular components
IP: Immunoprecipitation
